# Using machine learning models to predict oxygen saturation following ventilator support adjustment in critically ill children: A single center pilot study

**DOI:** 10.1371/journal.pone.0198921

**Published:** 2019-02-20

**Authors:** Sam Ghazal, Michael Sauthier, David Brossier, Wassim Bouachir, Philippe A. Jouvet, Rita Noumeir

**Affiliations:** 1 Department of health information analysis, École de Technologie Supérieure (ÉTS), Montreal, Quebec, Canada; 2 Department of Pediatrics, Sainte-Justine Hospital, Montreal, Quebec, Canada; 3 LICEF research center, TÉLUQ University, Montreal, Quebec, Canada; University of California San Francisco, UNITED STATES

## Abstract

**Background:**

In an intensive care units, experts in mechanical ventilation are not continuously at patient’s bedside to adjust ventilation settings and to analyze the impact of these adjustments on gas exchange. The development of clinical decision support systems analyzing patients’ data in real time offers an opportunity to fill this gap.

**Objective:**

The objective of this study was to determine whether a machine learning predictive model could be trained on a set of clinical data and used to predict transcutaneous hemoglobin oxygen saturation 5 min (_5min_ SpO_2_) after a ventilator setting change.

**Data sources:**

Data of mechanically ventilated children admitted between May 2015 and April 2017 were included and extracted from a high-resolution research database. More than 776,727 data rows were obtained from 610 patients, discretized into 3 class labels (< 84%, 85% to 91% and c92% to 100%).

**Performance metrics of predictive models:**

Due to data imbalance, four different data balancing processes were applied. Then, two machine learning models (artificial neural network and Bootstrap aggregation of complex decision trees) were trained and tested on these four different balanced datasets. The best model predicted SpO_2_ with area under the curves < 0.75.

**Conclusion:**

This single center pilot study using machine learning predictive model resulted in an algorithm with poor accuracy. The comparison of machine learning models showed that bagged complex trees was a promising approach. However, there is a need to improve these models before incorporating them into a clinical decision support systems. One potentially solution for improving predictive model, would be to increase the amount of data available to limit over-fitting that is potentially one of the cause for poor classification performances for 2 of the three class labels.

## Introduction

In case of respiratory failure, mechanical ventilation supports the oxygen (O_2_) diffusion into the lungs and the carbon dioxide (CO_2_) body removal. As an expert in mechanical ventilation cannot reasonably be expected to be continuously present at the patient’s bedside, specific medical devices aimed to help in ventilator settings adjustments may help to improve the quality of care [1]. Such devices are developed using algorithms either based on medical reasoning that adapt ventilator settings in real time based on patients’ characteristics [2, 3] or based on physiologic models that simulate cardiorespiratory responses to mechanical ventilation settings modifications [4]. The first ones are not accurate enough to be used widely in clinical practice, especially in children, and the latter are not validated for this indication. Both algorithms do not learn from ever-growing sets of clinical research data that could potentially improve their performances. To overcome this drawback, another avenue is the development of algorithms using artificial intelligence to provide caregivers with support in their decision-making tasks.

Among the vital parameters, transcutaneous hemoglobin saturation oxygen (SpO_2_) is monitored continuously at the bedside in intensive care and must be maintained in an adequate range to insure tissue oxygenation. In mechanically ventilated patients, when SpO_2_ is low, either FiO_2_ or ventilation pressures/volume are increased.

In this retrospective study, we assessed machine learning methods to predict the classification (normal, low or critically low) SpO_2_ of mechanically ventilated children after a ventilator setting change using a high-resolution research database. Such a modelling will help caregivers for the prescription of ventilator settings i.e. the caregiver will use the model to predict the effect of a ventilator setting change on SpO_2_ and will apply this ventilator modification if satisfied of the predicted SpO_2_.

## Materials and methods

This retrospective study was conducted at Sainte-Justine Hospital, Quebec, Canada and included the data collected prospectively between May 2015 and April 2017 of all the children, less than 18 years old, admitted to the Pediatric Intensive Care Unit (PICU) and were mechanically ventilated with an endotracheal tube. Patients’ data were excluded if the patient was hemodynamically unstable defined as 2 or more vasoactive drugs delivered at the same time (ie., epinephrine, norepinephrine, dopamine or vasopressin) or with an uncorrected cyanotic heart disease defined by no SpO_2_ > 97% during all PICU stay. All the respiratory data from included patients were extracted from the PICU research database [5], after study approval by the ethics review board (ERB) of Sainte-Justine hospital (ERB study number 2017 1480).

### Prediction problem

**Fig 1:**
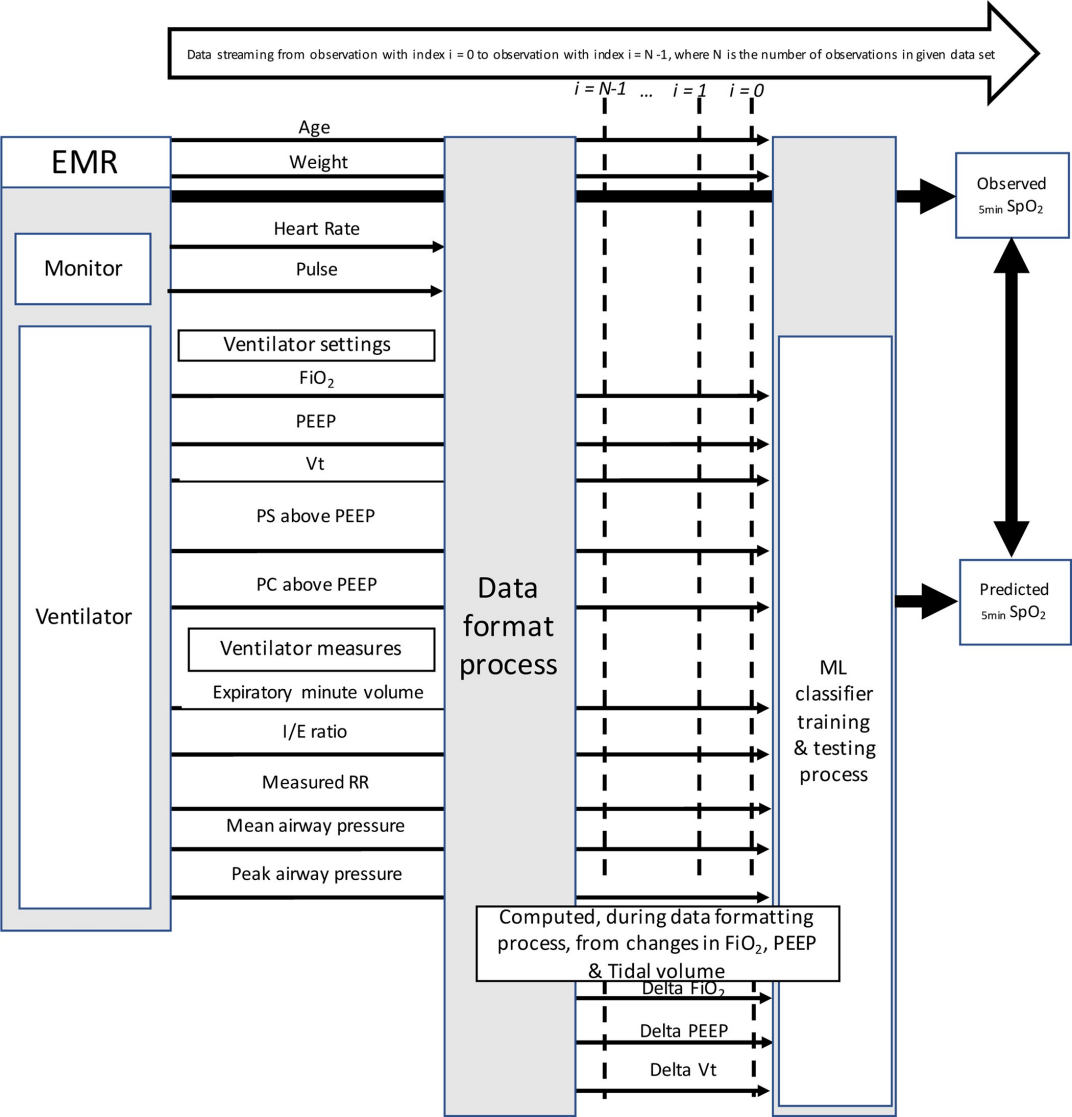
Schematic description of the items involved and analysis process. EMR: electronic Medical Record, FiO_2_: inspired fraction of Oxygen, Vt: tidal volume, PEEP: Positive end expiratory pressure, PS above PEEP: pressure support level Above PEEP, PC above PEEP: pressure control level above PEEP, I:E Ratio: inspiratory time over expiratory time, Measured RR: respiratory rate measured by the ventilator. _5min_SpO_2_: SpO_2_ observed 5 min after PEEP, FiO_2_, tidal volume, PS above PEEP, PC above PEEP change, ML: machine learning. Heart and pulse rate were only used to validate the database SpO_2_ value (see below and S1 File).

The predictive SpO_2_ class (prognostic class) was the SpO_2_ 5 minutes after a change of a ventilator setting. The delay of 5 min corresponded to the shortest period of time to reach a steady state after modification of a ventilator setting [6]. SpO_2_ levels at 5min were classified into three categories (Table 1). The thresholds were selected according to clinical value: a SpO_2_ < 92% is a target to increase oxygenation in mechanically ventilated children [7]. The critical level of 85% SpO_2_ is used as an alarm of severe hypoxemia in intensive care [8]. The success criteria for prediction was the ability of the model to predict the SpO_2_ category, 5min after a ventilator setting change *ie* delta in inspired fraction of Oxygen (ΔFiO_2_), delta in tidal volume, pressure support or pressure controlled (ΔVt, ΔPS or ΔPC) or delta in Positive end expiratory pressure set (ΔPEEP). The variables used in the model are detailed in Fig 1. These ventilator parameters were determined by an item generation-selection methods conducted by three physicians (PAJ, MS, DB). The resulting items are presented in Fig 1 within their sources, means of extraction and a schematic of the main components of the study.

**Table 1:** Definition of SpO_2_ class labels.

SpO_2_ classification	SpO_2_ range (%)	Rows number (n)
1	< 84	17,112
2	85 to 91	29,869
3	92 to 100	729,746

### Data preparation for model building

The data were extracted from a research database approved by the ethics committee of Sainte-Justine Hospital (database ERB number 2016–1210, 4061). The data extracted from the research database needed: (1) to remove erroneous data due to disconnection of the patient from the ventilator or the monitor, or due to transient interventions such as suctioning; (2) to remove the rows at which no ventilator setting variables was modified; (3) to adapt data format for classifier training. The methodology to format the data is described in S1 File. In summary, we first transformed the data from the linear format into a table, where the clinical variables are the column labels and the patient codes and storing times are the row labels. Since the readings for the various variables involved are not all set at the same frequency, the data for the different variables were aligned along the rows time-steps. Then, only the rows at which at least one of the setting variables is modified were preserved in the data file. The rows with change in “FiO_2_ Setting” more than 0.2 were excluded, to remove increase of FiO_2_ to 1 when suctioning. For each row, the target variable is added by binning the data of variable “SpO_2_ in 5 min” into three classes (Table 1). The binning of the target variable data into three classes allows for better classification performance. For all time-steps, SpO_2_ values were validated and kept in the database if heart rate (HR) from monitors in each row was within ± 10 bpm the HR from the pulse oximeter. All rows containing HR readings which do not respect this condition were removed.

The number of patients included was 610 mechanically ventilated children and the total number of rows according to SpO_2_ classification is specified in Table 1. We randomly distributed the number of rows between the training and test databases, without considering the number of patients in each dataset.

### Data balancing

The data analysis showed a severe imbalance with most SpO_2_ at 5min above 92%. This is logical as caregivers want to maintain SpO_2_ in normal range during child PICU stay. In such condition, the classifier learns the majority class label (class 3) (Table 1) but doesn’t learn the minority class labels (class 1 and 2) [9]. As, the data balancing process aims to allow the classifier to learn from all class equally, a combination of down-sampling and up-sampling techniques were included: to balance the three classes of the data involved, a down-sampling of the SpO_2_ class 3 using TOMEK algorithm [10] and an over-sampling of SpO_2_ class 1 and 2 using Synthetic Minority Oversampling Technique (SMOTE) [11] were performed.

The down-sampling process was made up of the following steps: (1) TOMEK algorithm was used to detect TOMEK links throughout the whole dataset, for all three classes, and removed them. TOMEK links are the links between any two observations considered nearest neighbors, but which belong to different classes [9], (2) points remainders removed are selected at random.

The creation of synthetic data points by SMOTE can be formulated as follows:

$$x_{syn}=x_{i}+(x_{knn}-x_{i})\times \delta$$

In this equation, *x*_*syn*_ represents the synthetic data point. The variables *x*_*i*_ and *x*_*knn*_ are respectively the original instance, and the nearest neighbor data point which is randomly picked among the *k* nearest neighbors. The random number *δ* is generated in [0,1] to determine the position of the created synthetic data point along a straight line joining the original data point *x*_*i*_ and its chosen nearest neighbor *x*_*knn*_.

To study which data balancing method provided the more accurate algorithm, four datasets were produced via four different balancing procedures, involving different combinations of data balancing techniques (Table 2).

**Table 2:** Descriptions of the four balancing procedures. The training/test split was done on the number of data samples.

dataset 1	dataset 2	dataset 3	dataset 4
**Training set**: 975,036 samples **Test set:** 193,528 samples **Class Balancing:** TOMEK applied to dataset (before dataset has been split into training & test set) to remove tomek links, random undersampling applied to class 3 once dataset is split into training and testing sub-sets, then SMOTE applied to classes 1 and 2 to make their cardinalities equal to that of class 3 (325,012).	**Training set**: 2,293,119 samples **Test set:** 201,926 samples **Class Balancing:** SMOTE applied to classes 1 & 2 to make their cardinalities equal to that of class 3 (764,373).	**Training set**: 487,464 samples **Test set:** 106,028 samples **Class Balancing:** TOMEK applied to dataset (before dataset has been split into training & test set) to remove tomek links, random undersampling applied to class 3 once dataset is split into training and testing sub-sets, then SMOTE applied to classes 1 and 2 to make their cardinalities equal to that of class 3 (162,488).	**Training set**: 1,462,503 samples **Test set:** 281,028 samples **Class Balancing** TOMEK applied to dataset (before dataset has been split into training & test set) to remove tomek links, random undersampling applied to class 3 once dataset is split into training and testing sub-sets, then SMOTE applied to classes 1 and 2 to make their cardinalities equal to that of class 3 (487,501).

### Predicted SpO_2_ model construct

To identify the best machine learning classification method, we tested two classification models: artificial neural network and bagged complex decision trees, on the four balanced training datasets.

#### Artificial Neural Network (ANN)

Once the data has been pre-processed, a machine learning predictive model was trained on a sub-set of labeled training data. The model is then used to predict the target variable values on a testing subset where the class labels are hidden. We used Artificial Neural Networks (ANN) to make predictions of the SpO_2_ variable, based on the values of the other variables of interest. Through the function approximation that the ANN performs, it is possible to make predictions of SpO_2_ variable, based on the input data. The outputs are the probability for each of the 3 class where the sum of their probabilities is 1.

The ANN is learned from training data, using the backpropagation algorithm [12] and is tested on a test set made of the remaining rows of data to validate the generalization of the model. The learning algorithm runs through all the rows of data in the training data set and compares the predicted outputs with the target outputs found in the training data set. The weights are adjusted via supervised learning, in a manner to minimize the error of predicted SpO_2_ vs target SpO_2_. The process is repeated until the error is minimized.

The ANN classifier was implemented through cycles of forward propagation followed by backward propagation through the network’s layers. The backpropagation algorithm is used for performance optimization. For detailed information see S2 File.

#### Bootstrap aggregation of complex decision trees

Bootstrap aggregating (acronym: bagging) was proposed by L Breiman in 1994 to improve classification by combining classifications of randomly generated training sets [13]. Bagging allows for the creation of an aggregated predictor via the use of multiple training sub-sets taken from the same training set. Let **(*T^i^*)** denote the replicate training sub-sets bootstrapped from the training set ***T***. These replicate sub-sets each contain *N* observations, drawn at random and with replacement from ***T***. For each of these sub-sets of *N* observations, a prediction model, or classifier, is created. The computational model used for bagging was complex decision trees. This means that, for each bootstrapped sub-set of training data, a complex decision tree is trained and thus a classifier is created. If *i = 1*, *…*, *n*, then *n* classifiers are created through the bagging process.

A decision tree is a flowchart computational model which can be used for both regression, as well as classification problems. Paths from the root of the tree to its various leaf nodes go through decision nodes in which decision rules are applied in a recursive manner, based on values of input variables. Each path represents an observation (***X***, *y*) = (*x*_*1*_, *x*_*2*_, *x*_*3*_, *…*, *x*_*n*_, *y*), where the label assigned to the target *y* is given in the leaf node, at the end of the path *i*.*e*. classification [14].

The measure used to build sub-trees was the gini index (see infogain.doc for details). We tested the BACDT model using 30, 50 and 70 decision trees.

In the aim of maximizing the model’s generalization capability during the training process, the Bagged Complex Trees’ performance is tested via *k*-fold cross-validation. A value *k* = 10, which is common practice, was used in this study for both the complex decision trees and ANN. The training using *k*-fold cross-validation is carried out as described below:

The data-set is first divided into two parts; the training-set and the test-set. The training of the “Bagged” Complex Trees includes a k-fold cross-validation, which is performed as follows:

- Randomly partition the data-set into k equal-sized subsets (folds).
- For each of the k equal-sized subsets:
  ○ Train/fit the model on the elements contained in the other (k-1) subsets.
  ○ Test the model’s accuracy on the given subset.
- Iterate over the k subsets, until each one has been used once for testing the model’s performance during its training.
- The training validation score consists of the average score obtained by validating the model on all k subsets.

The mathworks Matlab R2016b Machine Learning toolbox was used for the creation of the ensemble of Bagged complex trees model. The ANN classifiers were implemented using the Scikit-Learn package within the Python programming language [http://scikit-learn.org].

### Classifiers performances assessments

If the model outputted a predicted probability >0.9 for a given class, then the predicted class was considered positive. We evaluated the performances of the classifiers based on the metrics including ROC curves, average accuracy, precision (ratio of all correct classifications for class *i* to all instances labeled as class label *i* by the model), recall (ratio of the number of instances classified in class label *i* to the number of true class *i* labels) and F score (single measure of classification performance of the model used), see S2 File for further details [15].

## Results

**Fig 2:**
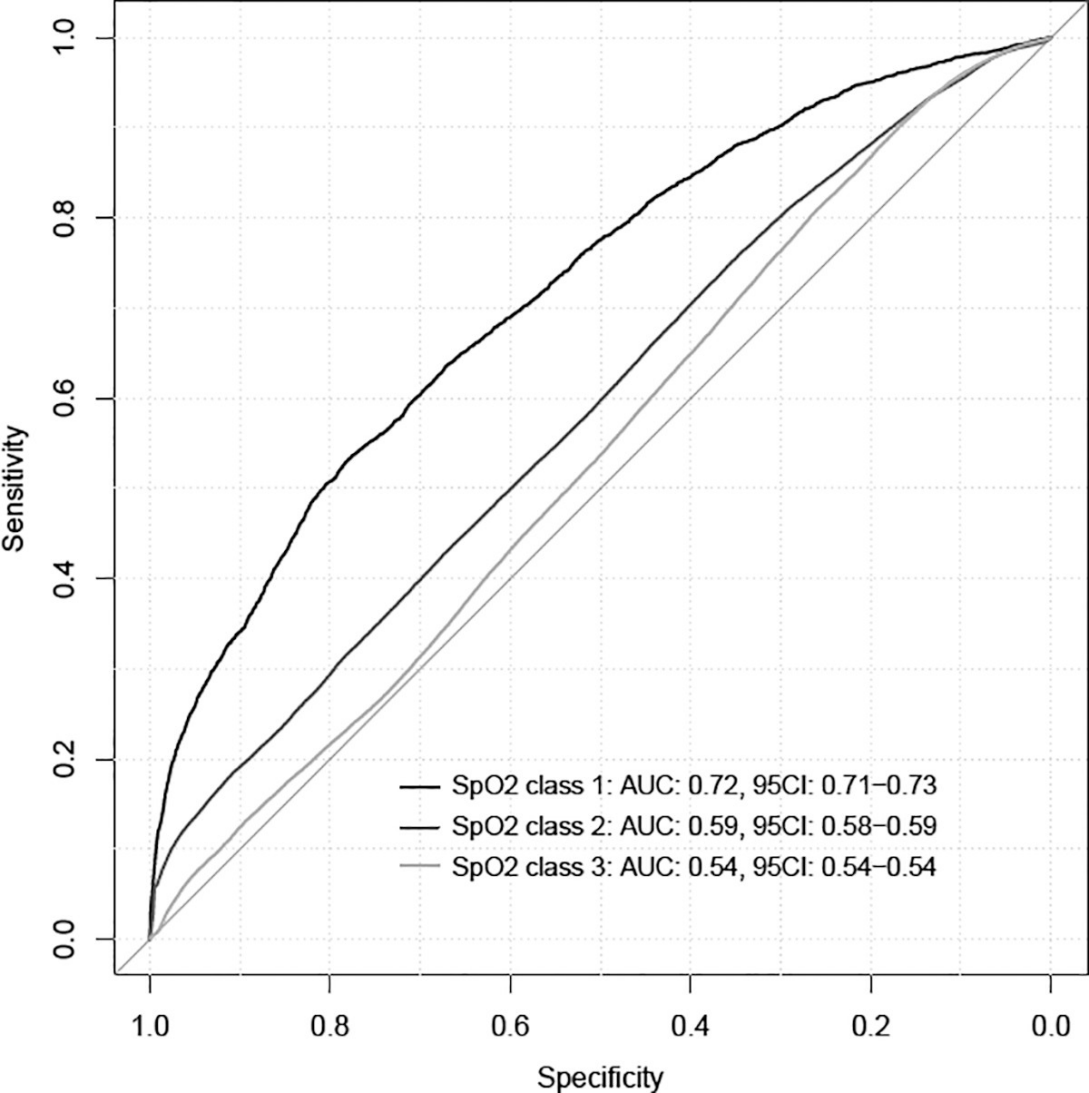
ROC curve for each SpO_2_ prediction at 5 min following a ventilator setting change of the best predictive model (bootstrap aggregation of complex decision trees (BACDT) classifiers on Test Dataset 3). Class 1: _5 min_SpO_2_ < 84%, class 2: _5 min_SpO_2_ between 85% and 91%, class 3: _5 min_SpO_2_ between 92% and 100%. AUC: area under the curve, 95IC: 95% confidence interval.

The number of patients included was 610 mechanically ventilated children with a median duration of ventilation of 33hrs (1^st^ quartile: 6.5hr and 3^rd^ quartile: 116.9 hr), similar to a previous study [16]. In the 776,727 ventilator settings modifications (Table 1), 98% of the ventilator settings modifications were FiO_2_ setting changes. The performances of the two machine learning classifiers to predict SpO_2_ at 5 min after a ventilator setting change (*ie* FiO_2_, PEEP, Vt/Pressure support or pressure controlled above PEEP) was developed on four different balanced training datasets and assessed on four different balanced test datasets (see Table 2). In Fig 2 and Table 3, we report the performances of these two classifiers. Using the classification performance metrics, the bagged trees classifier trained on dataset #3 has yielded the best classification performance on the test sets (Table 3) and was the predictive model retained. The ROC curves are shown in Fig 2 with area under the curves below 0.75 for all class.

**Table 3:** Performance of artificial neural networks (ANN) and bootstrap aggregation of complex decision trees (BACDT) classifiers for SpO_2_ prediction at 5 min following a ventilator setting change, on test datasets (see Table 2). Avg/total: average accuracy of total classification values. In italics is the performance of the best predictive model obtained among the eight tested.

Balanced datasets	_5min_SpO_2_ class	ANN			BACDT		
		Precision	Recall	F-score	Precision	Recall	F-score
**Dataset 1**	1	0.12	0.70	0.21	0.80	0.76	0.78
	2	0.16	0.43	0.23	0.61	0.56	0.59
	3	0.96	0.67	0.79	0.97	0.98	0.97
	Avg/total	0.88	0.65	0.73	0.94	0.94	0.94
**Dataset 2**	1	0.09	0.72	0.16	0.77	0.72	0.74
	2	0.09	0.47	0.16	0.57	0.53	0.55
	3	0.98	0.70	0.81	0.98	0.99	0.98
	Avg/total	0.93	0.69	0.78	0.96	0.97	0.97
**Dataset 3**	1	0.16	0.68	0.25	**0.80**	**0.76**	**0.78**
	2	0.26	0.42	0.33	**0.67**	**0.62**	**0.65**
	3	0.92	0.60	0.72	**0.95**	**0.96**	**0.96**
	Avg/total	0.80	0.58	0.65	**0.91**	**0.91**	**0.91**
**Dataset 4**	1	0.09	0.69	0.16	0.80	0.74	0.77
	2	0.12	0.47	0.19	0.58	0.54	0.56
	3	0.97	0.68	0.80	0.98	0.98	0.98
	Avg/total	0.92	0.67	0.76	0.96	0.96	0.96

### Impact of hidden layers for ANN and number of complex trees for BACDT on performance

For the artificial neural network, the variation of the number of hidden layers and number of neurons per hidden layer did not seem to have a significant effect on the model’s classification performance (Table 4). As for the Bagged complex trees, the variation of the number of complex trees did not yield significant changes in classification performance (Table 5). The number of decision trees used in best BACDT model was 50.

**Table 4:** Absence of impact on performance of the increase of neurons and hidden layers for artificial neural network (ANN). Example of the performance assessed by the F score on the balanced test dataset 3 (see Table 2).

ANN										
Error minimization algorithm		Stochastic Gradient-Descent (SGD)								
Activation function		Logistic Sigmoid								
Regularization		No								
Nb hidden layers (n)		1			2			3		
Neurons/hidden layer (n)		10	50	100	10	50	100	10	50	100
**F-score**	_5min_SpO_2_ class 1	0.25	0.25	0.25	0.25	0.25	0.25	0.22	0.22	0.19
	_5min_SpO_2_ class 2	0.33	0.33	0.33	0.33	0.33	0.33	0.33	0.33	0.32
	_5min_SpO_2_ class 3	0.72	0.72	0.72	0.72	0.72	0.72	0.69	0.69	0.69

**Table 5:** Absence of impact on performance of the number of complex trees for bootstrap aggregation of complex decision trees (BACDT). Example of the performance assessed by the F score on the balanced test dataset 3 (see Table 2).

		BACDT	
		n = 30	n = 50
**F-score**	_5min_SpO_2_ class 1	0.78	0.78
	_5min_SpO_2_ class 2	0.65	0.65
	_5min_SpO_2_ class 3	0.96	0.96

## Discussion

This single center pilot study using machine learning predictive model resulted in a predictive model with a poor accuracy (area under the ROC curves < 0.75). The comparison of machine learning models showed that bagged complex trees was the best approach. However, the model was of limited value for to predict SpO_2_ below 92%.

In agreement with previous studies regarding bagging being a better method for medical data classification, tree Bagging fared better than the artificial neural network [13]. The gap in performance between the training and testing confusion matrices in the case of bagged trees model (data not shown) seems to indicate that, although the bagged trees model was capable of learning very well from the data, there’s still room for improvement in the generalization. The SMOTE algorithm is designed in such a way that should theoretically not affect the generalization of the trained model. However, in cases of extreme data imbalance, as in this study, the over-sampling of minority class label is also likely to be extreme. This may render the data space of this class relatively dense with respect to the rest of the data made up of real data points. This may potentially explain the classification model’s relatively poor generalization for _5min_SpO_2_ class “1” and “2”. Also, since SMOTE generates synthetic data points by interpolating between existing minority class instances, it can increase the risk of over-fitting when classifying minority class labels, since it may duplicate minority class instances but this needs to be further investigated.

The strengths of this study include a large clinical database of mechanically ventilated children with more 776,727 rows. In a recent similar study in PICU, 200 patients were included with 1,150 rows [17]. However, the volume of data is clearly insufficient. To use such machine learning predictive models both for low SpO_2_ class and for ventilator setting modification such as PEEP. The pediatric intensive care community needs to combine multicenter high resolution database to increase the datasets. In addition, children data could be pooled to neonatal and adult intensive care data, when possible, such as MIMIC III database in specific clinical analysis[18]. The other strength is the process used to transform the data into a usable format and to correct a variety of artifacts (see S1 File). In health care, there is a significant interest in using clinical databases including dynamic and patient-specific information to develop clinical decision support algorithms. The ubiquitous monitoring of critical care units’ patients has generated a wealth of data that creates many opportunities in this domain. However, when developing algorithms, such as transport or finance, data are specifically collected for research purposes. This is not the case in healthcare where the primary objective of data collection systems is to document clinical activity, resulting in several issues to address in data collection, data validation and complex data analysis [19]. As detailed in S1 File, a significant amount of effort is needed, when data have been successfully archived and retrieved, to transform the data into a usable format for research.

This study has several limitations. First, the limited row number in low SpO_2_ levels reduced the SpO_2_ classification for machine learning predictive model to three clinically relevant classes. SpO_2_ is a continuous variable and the use of three class is probably insufficient [20, 21]. Instead of the classification model, the next step could be to test regression models’ performance. Second, SpO_2_ was predicted at 5min after ventilator setting change, a clinically relevant delay. However, the delay between ventilator setting change and oxygenation steady state is not well defined and vary from 1 to 71 minutes according to the parameter set (FiO_2_, PEEP or other parameters that change mean airway pressure) and clinical conditions studied [17, 22, 23]. This needs further research and probably more sophisticated clinical decision support systems using machine learning predictive models should consider these factors. Third, we excluded hemodynamic unstable patients using a treatment criteria (≥ 2 vasoactive drugs infused) because this condition decreases pulse oximeter reliability [24, 25]. The validation and electronic availability of reliable markers of hemodynamic instability in children such as plethysmographic variability indices could be helpful [26]. Finally, based on the classification approach taken, we didn't stratify the number of unique patients whose data were used for training versus testing, but only the number of instances for train versus test. The median duration of ventilation in our PICU is 33 hours, the medical conditions are numerous and the weaning phase where lung condition is almost the same among children represents 50% of the mechanical ventilation duration [16]. By random, the number of unique patient in the training and validation dataset is proportional to the whole population and reflects the whole PICU population studied. If we had determined a given number of patient per training and validation, we probably should also ned to dispatch the medical condition, the duration of ventilation, the underlying medical conditions. To address this problem, we included in the model variables that characterize the patient and lung severity at a given time including age, weight and mean airway pressure (see Fig 1).

## Conclusion

This pilot study using machine learning predictive model resulted in an algorithm with poor accuracy. We have proposed a method to apply supervised machine learning algorithms to extract knowledge from large amounts of patient mechanical ventilation data. Our method aimed at predicting the behavior of SpO_2_, based on ventilator setting changes made by the clinician and other clinical variables. To do that, we have exploited large amounts of data from a PICU research database and proposed a data formatting process which creates datasets that can be used for supervised training. The comparison of machine learning models showed the use of ensembles of bagged complex trees to be a promising approach. As for future work, various approaches and methods may be considered, in the aim of improving prediction of SpO_2_ classification, or level prediction in the case of regression models. One potentially viable solution for improving predictive models would be to use a greater amount of data. Although this could not be considered a warrant for better classifier robustness, it will decrease the need of a data balancing process and may be a relatively simple approach to be considered in future work. This will require a multicenter pediatric intensive care high resolution databases. For the moment, the study presents a model that predicts SpO_2_ using known setting changes made by the clinician, as well as the other clinical data that the clinicians involved in the study deemed relevant for SpO_2_ prediction. However, it is hoped that this predictive model will be incorporated in a larger Clinical Decision Support System to assist PICU clinicians in making decisions about required setting changes, based on the range in which SpO_2_ and other parameters (PaCO_2_, hemodynamic status, …) are to be maintained.

## Supporting information

S1 File Data formatting process. (DOCX)

S2 File Performance tests used in the machine learning models to predict oxygen saturation following ventilator support adjustment in critically ill children. (DOCX)

## References

[1] Jouvet P, Hernert P, Wysocki M. Development and implementation of explicit computerized protocols for mechanical ventilation in children. Annals of intensive care. 2011;1(1):51 10.1186/2110-5820-1-51 22189095 PMC3261103

[2] Rose L, Schultz M, Cardwell C, Jouvet P, McAuley D, Blackwood B. Automated versus non-automated weaning for reducing the duration of mechanical ventilation for critically ill adults and children: a cochrane systematic review and meta-analysis. Crit Care. 2015;19:48 10.1186/s13054-015-0755-6 25887887 PMC4344786

[3] Jouvet P, Eddington A, Payen V, Bordessoule A, Emeriaud G, Gasco R, et al A pilot prospective study on closed loop controlled ventilation and oxygenation in ventilated children during the weaning phase. Crit Care. 2012;16(3):R85 10.1186/cc11343 22591622 PMC3580628

[4] Flechelles O, Ho A, Hernert P, Emeriaud G, Zaglam N, Cheriet F, et al Simulations for mechanical ventilation in children: review and future prospects. Crit Care Res Pract. 2013;2013:943281 10.1155/2013/943281 23533735 PMC3606750

[5] Brossier D, El Taani R, Sauthier M, Roumeliotis N, Emeriaud G, Jouvet P. Creating a High-Frequency Electronic Database in the PICU: The Perpetual Patient. Pediatr Crit Care Med. 2018;19(4):e189–e98. 10.1097/PCC.0000000000001460 29406373

[6] Cakar N, Tu014Frul M, Demirarslan A, Nahum A, Adams A, Akýncý O, et al Time required for partial pressure of arterial oxygen equilibration during mechanical ventilation after a step change in fractional inspired oxygen concentration. Intens Care Med 2001;27(4):655–9. 10.1007/s001340100900 11403067

[7] Pediatric Acute Lung Injury Consensus Conference G. Pediatric acute respiratory distress syndrome: consensus recommendations from the Pediatric Acute Lung Injury Consensus Conference. Pediatr Crit Care Med. 2015;16(5):428–39. 10.1097/PCC.0000000000000350 PMC5253180 25647235

[8] Les recommendations des experts de la SRLF. Le monitorage et les alarmes ventilatoires des malades ventilés artificiellement. Réanim Urgences. 2000;9:407–12.

[9] Chawla N, Japkowicz N, A. Kotcz A. Editorial: special issue on learning from imbalanced data sets. ACM SIGKDD Explorations Newsletter. 2004;6:1–6.

[10] Elhassan T, Aljurf M, Al-Mohanna F, Shoukri M. Classification of Imbalance Data using Tomek Link (T-Link) Combined with Random Under-sampling (RUS) as a Data Reduction Method. Journal of Informatics and Data Mining. 2016;1:1–12.

[11] Chawla N, Bowyer K, Hall L, Kegelmeyer W. SMOTE: Synthetic Minority Over-sampling Technique. Journal of Artificial Intelligence Research. 2002;16:321–57. 10.1613/jair.953

[12] Gnana Sheela K, Deepa S. Review on methods to fix number of hidden neurons in neural networks. Mathematical Problems in Engineering. 2013;2013:11 10.1155/2013/425740.425740

[13] Breiman L. Bagging predictors. Berkeley: University of California, Statistics Do; 1994 421.

[14] Safavian S, Landgrebe D. A survey of decision tree classifier methodology. IEEE Transactions on Systems, Man, and Cybernetics. 1991;21(3):660–74. 10.1109/21.97458

[15] Sokolova M, Lapalme G. A systematic analysis of performance measures for classification tasks. Information Processing & Management. 2009;45(4):427–37. 10.1016/j.ipm.2009.03.002

[16] Payen V, Jouvet P, Lacroix J, Ducruet T, Gauvin F. Risk factors associated with increased length of mechanical ventilation in children. Pediatr Crit Care Med. 2012;13(2):152–7. 10.1097/PCC.0b013e3182257a24 21760567

[17] Smallwood CD, Walsh BK, Arnold JH, Gouldstone A. Equilibration Time Required for Respiratory System Compliance and Oxygenation Response Following Changes in Positive End-Expiratory Pressure in Mechanically Ventilated Children. Crit Care Med. 2018;46(5):e375–e9. 10.1097/CCM.0000000000003001 29406422

[18] Johnson AE, Pollard TJ, Shen L, Lehman LW, Feng M, Ghassemi M, et al MIMIC-III, a freely accessible critical care database. Sci Data. 2016;3:160035 10.1038/sdata.2016.35 27219127 PMC4878278

[19] Johnson AE, Ghassemi MM, Nemati S, Niehaus KE, Clifton DA, Clifford GD. Machine Learning and Decision Support in Critical Care. Proceedings of the IEEE Institute of Electrical and Electronics Engineers. 2016;104(2):444–66. oi: 10.1109/JPROC.2015.2501978. 10.1109/JPROC.2015.2501978 PMC5066876 27765959

[20] Girardis M, Busani S, Damiani E, Donati A, Rinaldi L, Marudi A, et al Effect of Conservative vs Conventional Oxygen Therapy on Mortality Among Patients in an Intensive Care Unit: The Oxygen-ICU Randomized Clinical Trial. JAMA. 2016;316(15):1583–9. 10.1001/jama.2016.11993 27706466

[21] Pannu SR, Dziadzko MA, Gajic O. How Much Oxygen? Oxygen Titration Goals during Mechanical Ventilation. Am J Respir Crit Care Med. 2016;193(1):4–5. 10.1164/rccm.201509-1810ED 26720783

[22] Tugrul S, Cakar N, Akinci O, Ozcan PE, Disci R, Esen F, et al Time required for equilibration of arterial oxygen pressure after setting optimal positive end-expiratory pressure in acute respiratory distress syndrome. Crit Care Med. 2005;33(5):995–1000. 15891327 10.1097/01.ccm.0000163402.29767.7b

[23] Fildissis G, Katostaras T, Moles A, Katsaros A, Myrianthefs P, Brokalaki H, et al Oxygenation equilibration time after alteration of inspired oxygen in critically ill patients. Heart Lung. 2010;39(2):147–52. 10.1016/j.hrtlng.2009.06.009 20207275

[24] Salyer J. Neonatal and pediatric pulse oximetry. Respir care. 2003;48(4):386–96. 12667266

[25] Fouzas S, Priftis KN, Anthracopoulos MB. Pulse oximetry in pediatric practice. Pediatrics. 2011;128(4):740–52. 10.1542/peds.2011-0271 21930554

[26] Chandler JR, Cooke E, Petersen C, Karlen W, Froese N, Lim J, et al Pulse oximeter plethysmograph variation and its relationship to the arterial waveform in mechanically ventilated children. J Clin Monit Comput. 2012;26(3):145–51. 10.1007/s10877-012-9347-z 22407178

