# Supplementary material for: Using machine learning models to predict oxygen saturation following ventilator support adjustment in critically ill children: A single center pilot study
Source: PLoS One. 2019 Feb 20;14(2):e0198921. doi: 10.1371/journal.pone.0198921 (PMC6382156; doi:10.1371/journal.pone.0198921)
Supplement: S1 File — (DOCX) [file pone.0198921.s001.docx]

### **S1 File: Data formatting**

The extraction of data from the patient database produces data files with a format which doesn’t allow for classifier training. This is mainly because, in these initial data files, the respiratory variables are not separated in columns, which would become the input and output vectors during model training. Moreover, a significant proportion of rows in the initial files do not represent data which are measured within mechanical ventilation time intervals. Thus, it is necessary for the formatted files which are to be used for classifier training to be rid of any unnecessary rows, which do not contain mechanical ventilation readings. The classifiers used to predict the SpO_2_ values are built when mathematical models are trained on a set of data which displays the relevant variables in a table format. The table must be arranged as follows: the respiratory variables represent the labels of the various columns and the data storing times and patient codes represent the rows. The data formatting process described herein consists basically in making the data format machine learning-friendly. In this section, we present the steps, mostly established by the clinicians, for formatting and pre-processing the raw data to make it suitable for classifier training:

- **Read content of initial data file into a Python data frame.**

The data contained in the initial files are stored into data frames which are used to manipulate and preprocess the data.

- **Strip away the microseconds part from the data storing times in the initial files, as it is not contextually relevant.**

Following this step, the storing times are represented by: *“year:month:day:hours:minutes:seconds”*

- **Pivot the data that were stored in the data frames.**

This step transforms the data from the linear format in the initial files into a table, where the clinical variables are the column labels and the patient codes and storing times are the row labels.

- **Align the data of the variables in the pivoted table within mechanical ventilation time slots.**  Since the readings for the various variables involved are not all set at the same frequency, the data for the different variables are not aligned along the rows time-steps. Therefore, it is necessary to align the data readings for the various variables along any given row within a mechanical ventilation interval, to prepare the data to be used for classifier training. This data formatting step ensures the alignment of the data for “HR”, “SpO_2_”, “Pulse”, "Pressure Support Level Above PEEP" and "Pressure Control Level Above PEEP" variables with the data of the other variables, for any given time-step within mechanical ventilation time-slots.
- **Fill cells of “Tidal Volume Setting” variable with the values given by *“Expiratory Minute Volume” / “Measured Frequency” x 1000,* as per clinician`s requirement.**
  - **Drop rows with any empty cell(s).**  Once alignment of the data is completed, all rows containing empty readings are to be dropped to ensure that only time-slots of mechanical ventilation readings are preserved.
  - **Create three new variables which represent the changes made to the setting variables.** Run through all the rows in the data frame and, for any of the three setting variables, if the value at any time-step is NOT EQUAL to the value at the previous time-step, then compute the difference between the values at both time-steps and place the results in new columns, called “Delta FiO_2_ Setting”, “Delta PEEP Setting” and “Delta Tidal Volume Setting”. This step allows the creation of three new variables: “Delta FiO_2_ Setting”, “Delta PEEP Setting” and “Delta Tidal Volume Setting”. For each patient section, as per the patient code, whenever the readings show that at least one of the three setting variables is modified from one row to the next, the values of the differences are stored in the new columns created. This means that only the rows at which at least one of the setting variables is modified are to be preserved in the data file which will be used for classifier training.

Two conditions must be met for the execution of the last step:

- - - - The data of different patients should be treated separately, as per the patient codes which these data are grouped by. This ensures that the readings for different patients are not mixed up.
      - The change in “FiO_2_ Setting” should not exceed 20%, as per clinician’s requirement. When patients are mechanically ventilated, suctioning needs to be regularly performed to remove tracheal secretions. During suctioning, FiO_2_ is increased to 100%. To remove data due to this intervention that is not linked with a patient respiratory improvement or worsening, we excluded the data when change in “FiO_2_ Setting” exceeded 20%.
- **Copy the value of “SpO_2_” at the row 5 minutes following the current examined row, into the current row, in a column assigned to this variable. We used 5 min. as the minimum settling time for SpO_2_ following setting changes.**
- **Create a new column called “Binned SpO_2_” in the data frame and fill it with values as per the binning criteria in table 1. This variable is to be used as the target variable.**

The target variable is created by binning the data of variable “SpO_2_ in 5 min.” into three classes (Table 1). The binning of the target variable data into three classes allows for better classification performance, since it reduces the size of the range of values that the trained model would have to predict from. This naturally implies that it increases the amount of observations per target class label, which allows the classification model to extract more information per class label, during the training process.

- - **For all time-steps, verify the accuracy of HR readings by making sure that they are within ± 10 of “Pulse”.** All rows containing HR readings which do not respect this condition are dropped.
  - **All rows in data frame where “Peak Airway Pressure” ≤ 5 cmH_2_O are dropped.** When the patient is disconnected from the ventilator, “Peak Airway Pressure” drops below 5 cmH_2_O. As we were studying patient evolution during mechanical ventilation, we excluded such situations.
  - **Add “Age” and “Weight” data of all patients to the data frame.**  Using the Patient-specific data file and the data frame which represents the data file which is used to train the predictive model, the age and the weight of every patient are added to the data frame in which the data is being formatted. The weight and the age of each patient, at the time of undergoing mechanical ventilation, are inserted in their newly created columns, at the appropriate rows, in the data frame. The data frame containing the formatted data is copied into a comma-separated file (csv) file.

### Feature standardizing and scaling

The predictive model’s training/testing trials have been carried out both on standardized and on scaled input data. These data pre-processing steps were deemed necessary, since the input variables have ranges of values which are very dissimilar.

The standardization, or z-score normalization, transforms the various data vectors, ie., the variables, in such a way that they will have the properties of a standard normal distribution with *µ* = 0 and *σ* = 1. Standardizing the variables so that they are centered around 0 with a standard deviation of 1 is not only important when measurements that have different units are compared, but it is also a general requirement for many machine-learning algorithms, including ANNs. The standardization is performed, ie., the z-score is computed, for every observation ***x_i_*** of a variable ***X***, using the mean $\mu(\boldsymbol{X})$ of the variable and its standard deviation $\sigma(\boldsymbol{X})$.

The data rescaling, on the other hand, allows for the conversion of the different input variable ranges to a common range, namely [0,1].

Feature data rescaling is performed as follows:

$x_{i}=\frac{x_{i}- x_{min}}{x_{max}- x_{min}}$ (1)

In equation 1, *x_i_* is the feature value at observation *i*, *x_min_* and *x_max_* are the minimum and maximum values of feature ***X***, respectively. For the data involved in this study, feature rescaling yielded better results than feature standardizing on model training and testing performances. Therefore, all the results presented in this paper are obtained via training and testing of the classifiers on the rescaled data (eq. 1).
