# Supplementary material for: Using machine learning models to predict oxygen saturation following ventilator support adjustment in critically ill children: A single center pilot study
Source: PLoS One. 2019 Feb 20;14(2):e0198921. doi: 10.1371/journal.pone.0198921 (PMC6382156; doi:10.1371/journal.pone.0198921)
Supplement: S2 File — (DOCX) [file pone.0198921.s002.docx]

### **S2 File: Supplement on Methods used in the machine learning models to predict oxygen saturation following ventilator support adjustment in critically ill children**

1. The ANN classifier was implemented through cycles of forward propagation followed by backward propagation through the network’s layers. The backpropagation algorithm is used for performance optimization.

The backpropagation algorithm is used to help fine tune the ANN weights in the aim of finding an optimal estimate of a function that models the training data. In other words, it allows for finding a set of weights which minimizes the error of the ANN's output, which itself depends on the ANN inputs, weights and activation function used. The hidden layer neurons as well as the output neuron compute their respective outputs by feeding a weighted sum of the weights and the outputs of the previous layer neurons into the activation function. An error function is used to compute the ANN's error via comparison of the ANN output with the target. Applying the derivative of the error function in an iterative manner using the chain rule, the model would ideally find an optimal set of weights for ANN error minimization.

For a given number of classes K > 2, the cross-entropy error can be formulated as shown in eq. 1, where (***W_i_***)*_i_* is the matrix of weights between the neuron layers, *r_i_* is the target value. *y_i_* is the value generated by the ANN, ie., its output. In eq 1, i denotes the sample index in the arrays of predicted and target output variable. The cross-entropy loss is obtained via a summing over the product of the log of the predicted SpO_2_ values and the target SpO_2_ values.

$\text{E}^{\text{t}}\text{(}\text{(}\text{W}\text{i}\text{)}\text{i }\text{|}x^{t}\text{, }r^{t}\text{) = - }\sum_{i} r_{i}^{t}{\log y}_{i}^{t}$(1)

The outputs of the ANN are:

$$y_{i}^{t}= \frac{\exp w_{i}^{t}x^{i}}{\sum_{k} \exp w_{k}^{t}x^{t}} (2)$$

k is the class number. Using stochastic gradient-descent (SGD) for error minimization, the update rule for the ANN weights is:

$$\text{ ∆}w_{ij}^{t}=\eta\left( r_{i}^{t}- y_{i}^{t} \right)x_{j}^{t} \left( 3 \right)$$

In equation 3, η represents the learning rate. When SGD is used, this learning rate decreases as the error tends to a minimum. During ANN training, each observation, comprised of an input vector and a target output, is denoted (***x****^t^,* ***r****^t^*), with ***r****^t^* ϵ { “1”, “2”, “3” }.

2. Performance tests:

- **Precision**

$$Precision =\frac{\# True positives class i}{Total \# classifications for class i} (4)$$

The *Precision* (eq. 4) is the ratio of all correct classifications for class *i* to all instances labeled as class label *i* by the model. In a non-normalized confusion matrix, this would mean dividing the number of instances classified in class label *i* by the total of instances in column *i.*

- **Recall**

$$Recall =\frac{\# True positives class i}{Total \# observations class i} (5)$$

Recall is the ratio of the number of instances classified in class label *i* to the number of true class *i* labels. In a non-normalized matrix, this would require dividing the number of instances classified in class label *i* by the total of row *i*

- **F-score**

$$F-score=\frac{2}{\frac{1}{recall}+\frac{1}{precision}} (6)$$

## *The F-score provides a single measure of classification performance of the model used.*
